# Home living older adults' subjective perceptions, evaluation, and interpretations of various welfare technology: A systematic review of qualitative studies

**DOI:** 10.1016/j.puhip.2024.100470

**Published:** 2024-01-19

**Authors:** Zada Pajalic, Sofia Elisabeth G. Olsen, Annabel Hamre, Benedicte Sørensen Strøm, Celine Clausen, Diana Saplacan, Gunilla Kulla

**Affiliations:** a Faculty of Health Sciences Sustainable Healthcare and Welfare Technology (SHWT) VID Specialized University, Oslo, Norway; b Lovisenberg Diaconal University College, Oslo, Norway; c Oslo Municipality, Grorudhuset, Oslo, Norway; d Robotics and Intelligent Systems (ROBIN) Research Group, University of Oslo, Norway; e Faculty of Health Sciences, Western Norway University of Applied Sciences, Forde, Norway

**Keywords:** Home living, Older adults, Perception, Welfare technology

## Abstract

**Objectives:**

This paper aims to map home-living older adults' subjective perceptions, evaluations, and interpretations of various welfare technologies.

**Study design:**

Systematic literature review.

**Methods:**

The study was designed as a systematic literature review of qualitative studies. This systematic literature review was carried out according to the PRISMA statement and was prospectively registered in PROSPERO registration number CRD42020190206. The international electronic bibliographic databases included AMED, Academic, CINAHL, Cochrane Reviews, EMBASE, Google Scholar, MEDLINE via PubMed, Scopus, and Web of Science. The scientific evidence was synthesized using qualitative analysis. All aspects of the study method followed COREQ guidelines.

**Results:**

Following a detailed systematic search and screening of 1405 studies, 10 were included in the systematic review. The study shows that implementing Welfare Technology seems to prolong older adults' independent living in their own homes and was perceived as a complement to face-to-face contact with health care providers.

**Conclusions:**

This study indicated that older adults consider accepting Welfare Technology as it contributes to a sense of security and empowerment in their everyday lives.

## 1 Introduction

The growth in the old people population [1] puts pressure on health workers and welfare services [2]. Among the solutions is Welfare Technology (WT) (e.g. information communication technology, ICT) used in aim to support independent Activities of Daily Living (ADL) for the home living older adults [3]. In this review, we have chosen to use the overall concept of WT, which refers to digital technology used to maintain independence, maintain daily activities, security and participation in the event of a risk of impaired bodily functions [[4], [5], [6]]. Hoffman defines WT as technical assistance that contributes to independent living [7]. The introduction and use of WT [3] allows for the provision of more goal-oriented and practical care services, increases security, safety, independence, manageability of daily life, prolongs living at home, helps prevent fall accidents at home, and improves quality of life [[8], [9], [10], [11]].

Hoffman groups WT based on purpose and function into the following categories: 1) communication technology, 2) compensation technology that complements lost and impaired functions, 3) technology that helps with practical work, 4) disease surveillance, 5) distance therapy technology [7]. In addition, digital technology in health is classified into three levels, from A to C. Level A deals with services in the form of technology to the caregivers. Level B includes general health monitoring tools for users and caregivers. Level C deals with the technology used for preventive health work focusing on healthy behavioural changes [12,13]. According to Hoffman [7], few studies shed light on how different types of ICT provide exchange and benefit old adults who are traditionally not used to using it. He also wonders whether ICT achieves the desired goals and what its side effects and costs are. In the Nordic context, the notion of WT refers to technologies and solutions that are used by users, including old people and others, in their everyday life to improve their daily living, promote independence, and reduces hospitalization or prolongs independent living in their own home [3,5,6,10]. The concept of WT originally came from the healthcare sector and is strongly connected to adapting care and technology to specific users. Many studies provide an illustrative example of Universal Design (UD) and WT, who explained that restaurants and cinemas have long been designed to facilitate the entrance of individuals sitting in wheelchairs by making an accessible entrance through the back door [[14], [15], [16]]. The Nordic countries pride themselves on fostering the diversity of their population's considerable variation in function [8,14,17]. These individuals require independent living solutions that allow them to age in place but also active citizen participation in society [11,18]. This paper aimed to map home-living older adults' subjective perceptions, evaluations, and interpretations of various welfare technologies.

## 2 Methods

This systematic review [19] was carried out according to the PRISMA checklist and statement [20] and prospectively registered in PROSPERO [21] registration number CRD42020190206. The critical aspects of the study method were followed by using COREQ guidelines [22].

### 2.1 Ethics statement

In this study, no new empirical material has been collected. Therefore, we have not applied for formal ethical approval. Included studies have undergone ethical review.

### 2.2 Eligibility criteria

We chose to include studies with a qualitative design to investigate subjective human experiences, and attitudes.

#### 2.2.1 Inclusion criteria

The inclusion criteria were specified:

● Primary studies with qualitative design published in English.
● Home-living old persons (≥60 years) who are users of WT
● Use of WT in various ways (audio-visual monitoring (AL), WT, video communication, apps, computer software, telemedicine, ICT, sensors, wearable devices)

#### 2.2.2 Exclusion criteria

The exclusion criteria were specified:

● Not primary study with qualitative design
● Study protocols, abstracts and non-peer-review publications, books, position papers or state-of-the-art publications
● Wrong population
● Study context other than older people's homes

### 2.3 Information sources

**Fig. 1:**
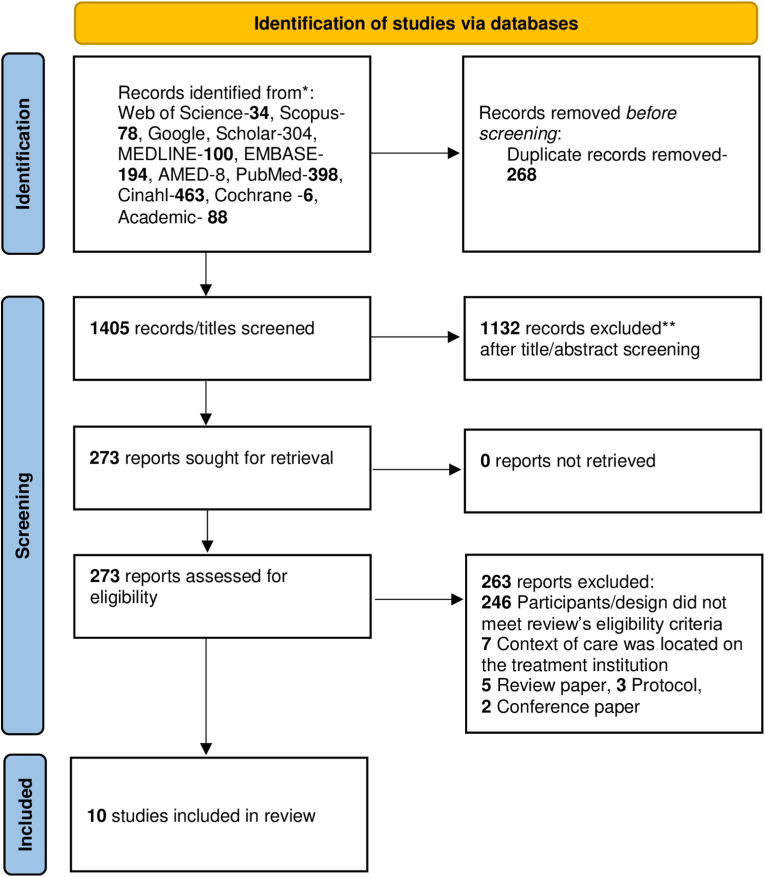
PRISMA flowchart and Table 4 around here.

Searches were performed in databases AMED, Academic, CINAHL, Cochrane Reviews, EMBASE, Google Scholar, and MEDLINE via PubMed, Scopus, and Web of Science (Fig. 1 PRISMA flowchart, and Table 1, Table 2).

**Table 1:** Example searching in databases.

Date	Database	Searching terms	Number of records found
11.11.2020	Web of Science	(ALL= (("older people" OR Aged OR Elderly) AND (home OR "independent living" OR "Community-Living") AND ("Welfare technology" OR "Health-technology") AND (qualitative))) AND LANGUAGE: (English) Indexes = SCI-EXPANDED, SSCI, A&HCI, ESCI Timespan = Last 5 years	34
11.11.2020	Scopus	ALL (“older people” OR aged OR elderly) AND (home OR “independent living” OR “Community-Living”) AND (“welfare technology”) AND (qualitative) AND (LIMIT-TO (PUBYEAR, 2021) OR LIMIT-TO (PUBYEAR, 2020) OR LIMIT-TO (PUBYEAR, 2019) OR LIMIT-TO (PUBYEAR, 2018) OR LIMIT-TO (PUBYEAR, 2017) OR LIMIT-TO (PUBYEAR, 2016) OR LIMIT-TO (PUBYEAR, 2015)) AND (LIMIT-TO (LANGUAGE, “English”))	104
11.11.2020	Google Scholar	“older people” AND (home OR “independent living”) AND “welfare technology” AND qualitative year 2015–2020	301

**Table 2:** Example searches in AMED.

for: 20 and 21 and 22 and 23	
Results: 2	
Database: AMED (Allied and Complementary Medicine) < 1985 to June 2020> Search Strategy:	
1	exp Aged/or aged.mp. (24056)
2	"Older adults" mp (3711)
3	Elderly.mp. (5824)
4	exp Aging/or Aging.mp. (4591)
5	assisted living.mp. (179)
6	living at home.mp. (238)
7	community living.mp. (293)
8	autonomy.mp. (1007)
9	exp independent living/or independent living.mp. (2472)
10	social participation.mp. (329)
11	exp Communication aids/or communication aids.mp. (484)
12	exp Medical informatics/or medical informatics.mp. (771)
13	exp Telemedicine/or telemedicine.mp. (1027)
14	telehealth.mp. (212)
15	tablets.mp. (247)
16	mobile phone.mp. (71)
17	smartphone.mp. (124)
18	Robotics/or robotics.mp. (585)
19	Assistive technology.mp. (545)
20	1 or 2 or 3 or 4 (28248)
21	5 or 6 or 7 or 8 or 9 or 10 (4216)
22	11 or 12 or 13 or 14 or 15 or 16 or 17 or 18 or 19 (3683)
23	randomized.mp. (12450)
24	20 and 21 and 22 and 23 (2) Search for: limit 28 to yr. = "2015–2020″

### 2.4 Search strategy

The search strategy following MeSH keywords were used, singly and in combination: older adults, community-living, e-health, medical informatics, health informatics, mobile health, health, telemedicine, telehealth, telecare, telemonitoring, e-learning, active and assisted living, self-help devices, technologies, assistive technology, everyday technology, own home, qualitative research design, descriptive, grounded theory, content analysis. Examples of searching strategies are illustrated in Tables 1–2.

### *2.5* Selection process

Selected titles were imported first into the reference management software EndNote [23] and then into the web tool Rayyan [24]. In total, 1673 titles were imported, and 268 duplicates were removed. After the first screening step, we excluded 1132 titles and found 273 titles that we deemed interesting for reading abstracts, full texts and discussion. This step resulted in an additional 255 records being excluded because they did not meet the inclusion criteria. Finally, we included ten eligible studies for quality assessment [[25], [26], [27], [28], [29], [30], [31], [32], [33], [34]].

### 2.6 Critical appraisal

The CASP checklist [35] was used to assess the quality of the articles selected for this review (Table 3) by several co-authors (ZP, GK, BSS) first independently and then as a group as we discussed our review findings until a consensus was reached.

**Table 3:** CASP assessment of included studies*.

Study	Q1	Q2	Q3	Q4	Q5	Q6	Q7	Q8	Q9	Q10	Sum
Bhattarai et al., 2020	1	1	1	1	1	1	1	1	1	1	10
Hanley et al., 2015, UK	1	1	1	1	1	1	1	1	1	1	10
Karlsen et al., 2018, Norway	1	1	1	1	1	1	1	1	1	1	10
Kärki & Kuusinen, 2016, Finland	1	1	1	1	1	1	1	1	1	1	10
Mortenson et al., 2016, Canada	1	1	1	1	1	1	1	1	1	1	10
Pol et al., 2016, Netherlands	1	1	1	1	1	1	1	1	1	1	10
Sánchez et al., 2019, Norway	1	1	1	1	1	1	1	1	1	1	10
Shulver et al., 2016, Australia	1	1	1	1	1	1	1	1	1	1	10
Thilo et al., 2016, Switzerland	1	1	1	1	1	1	1	1	1	1	10
Wu et al., 2019, Canada	1	1	1	1	1	1	1	1	1	1	10

*CASP Checklist (Yes = 1; Can't tell = X; No = 0).

10 questions to help you make sense of qualitative research: Q1 Was there a clear statement of the aims of the research? Q2 Is a qualitative methodology appropriate? Q3 Was the research design appropriate to address the aims of the research? Q4 Was the recruitment strategy appropriate to the aims of the research? Q5 Was the data collected in a way that addressed the research issue? Q6 Has the relationship between researcher and participants been adequately considered? Q7 Have ethical issues been taken into consideration? Q8 Was the data analysis sufficiently rigorous? Q9 Is there a clear statement of findings? Q10 How valuable is the research?.

### 2.7 Procedure of data extraction and compilation of data

Data was extracted from the articles and compiled into Table 4. Based on the purpose of the study, we categorized the results (Table 5, Table 6) from selected studies into the following themes: “*Welfare technology facilitates self-care and social contact”, “Welfare technology is perceived as helpful” and “Incorporating welfare technology into everyday life”*.

**Table 4:** Study characteristics data extraction, authors, country, CASP sore, technology type, length of use, results, the implication for practice.

Author, year, country, CASP score, title	Technologies	Length of use	Study design, nr. of participants, data collection, and analysis	Results	Implication for practice
Bhattarai et al., 2020, Australia, 10 Apps for pain self-management of older people's arthritic pain, one size doesn't ﬁt all: A qualitative study	App.	2-weeks	Qualitative study, 16 individual telephone interviews and thematic analysis	The app was judged to be valuable but must be adapted to the user's needs, the local context must be user-friendly, and the data collected in the app must be linked to the care providers so that they can adjust care, guidance, and support.	Not all older people find the app exciting or relevant, the app must be a link between the user and the caregiver to achieve the desired result; the app must be developed so that it can be used easily, and users must be involved in the design and development of the app
Hanley et al., 2015, UK, 10, Qualitative study of telemonitoring of blood glucose and blood pressure in type 2 diabetes	Tele-medicine	6-weeks	Qualitative study, 23 individual semi-structured face-to-face interviews and thematic analysis	Telemonitoring was perceived as feasible and accepted by users, but the problems were insufficient information on who takes the initiative or is responsible after blood sugar and blood pressure values have been reported into the system.	The technology must be refined; more evidence of its effect is needed for it to be adopted in clinical practice
Karlsen et al., 2018, Norway, 10, Caring by telecare? A hermeneutic study of experiences among older adults and their family caregivers	Tele-medicine	5–6 months	Qualitative hermeneutic research approach, 18 individual in‐depth semi-structured interviews and follow‐up interviews were conducted after 5–6 months	Increased security and increased independence	Telecare has a positive effect on security and independence but cannot cover all needs. Continuous assessment of what benefit telecare provides over time given changes in users' health status.
Kärki & Kuusinen, 2016, Finland, 10, How to live independently with or without technology?	ICT	NA	Qualitative study, 8 individual thematic interviews	The technology enables contact with others, can positively affect the quality of life but develops quickly and needs to be user-friendly for people with impaired physical or mental functions. Older people have different levels of skills to take advantage of ICT's benefits.	There is a need to support the need to learn the use of different ICT solutions in everyday life to increase the quality of life and feeling of self-determination.
Mortenson et al., 2016, Canada, 10, No place like home? Surveillance and what home means in old age	AAL	NA	Qualitative study, 27 individual semi-structured in-depth interviews	Ambient assisted living (AAL) contributes to the feeling of security in their own home but using AAL could be demoralizing and seen as a severe problem. Being watched by others was seen both as security and even as a loss of independence.	There is a need to understand better how AAL will affect the lives of residents being monitored. This is critical, so strategies can be implemented to prevent AAL from becoming an instrument of oppression rather than a technology of empowerment.
Pol et al., 2016, Netherlands, 10, Older People's Perspectives Regarding the Use of Sensor Monitoring in Their Home	Sensors	NA	Qualitative interpretative phenomenological study design 11 individual semi-structured interviews and interpretative phenomenological analysis	Sensors werepositively evaluated and seen as a strategy to enable independent living, increase safety, and remain activities. The health care professionals' continuous access to sensor data and use of the data for safety outweighed the privacy concerns	Sensor monitoring is an opportunity or strategy that can contribute to independent living and that will not disturb their natural way of living
Sánchez et al., 2019, Norway, 11, Older People's Attitudes and Perspectives Of Welfare Technology	WT	NA	Exploratory, qualitative approach 9 individual semi-structured, in-depth interviews and qualitative content analysis	Welfare technology was seen as a valuable solution to living as long as possible in their homes.	Welfare technology is a welcome a solution and promises many advantages for older people.
Shulver et al., 2016, Australia, 10 Well, if the kids can do it, I can do it’: older rehabilitation patients' experiences of telerehabilitation	Tele-medicine	8-weeks	Qualitative study, 13 individual qualitative interviews and thematic analysis	Telerehabilitation is convenient, promotes motivation and self-awareness, fosters positive therapeutic relationships, mastering technologies used by younger relatives is a valued aspect of telerehabilitation, and telerehabilitation does not replace traditional face-to-face rehabilitation therapies.	Telerehabilitation is acceptable the expanding use of technology to provide such services at a distance is workable and acceptable to older people and an available way of translating evidence into practice by increasing exercise dosage
Thilo et al., 2016, Switzerland, 10, Involvement of the end-user: an exploration of older people's needs and preferences for a wearable fall detection device – a qualitative descriptive study	Sensors, apps	NA	A qualitative descriptive study, Group interviews with 22 participants and qualitative descriptive analysis	The automatic and manual alerting functions of the bendable device and the sensor were made of waterproof material. They had optimal weight, and were wearable for 24 h in a row. They were welcomed. There were requirements regarding the reliability of the sensor that should determine its size.	The prototype of the fall detection device can be developed with a “need-driven” focus. End-users can contribute to the mock-up design stage in developing the device by indicating what matters to them. Older people's perception of activity, independence, and familiarity should be considered in developing a device.
Wu et al., 2019, Canada, 10, Using wearables and self-management apps in patients with COPD: a qualitative study	Wearable device and app	NA	Qualitative study, 14 individual semi-structured interviews and inductive thematic analysis	Wearable devices and self-management apps were viewed favourably with significant potential to help manage a health condition, maintain control of the information, make connections with the data, and be alerted when a possible exacerbation occurs.	Involving people with COPD in the design will be crucial to ensure critical components to aid self-management are effective and ensure barriers are addressed.

**Table 5:** Example of meaning units, condensed meaning units and codes.

Meaning units (text taken from the results of included studies)	Condensed meaning units	Codes
Apps are valuable self-management tool, but they do have the potential for harm. Participants perceived that the app was a valuable platform to access pain self-management resources, information, and instruction(s). Participants appreciated the accessibility of an app that contained a range of helpful pain self-management instructions to simplify one's everyday living with arthritis. I have quite a lot of arthritis in my fingers … and gripping things is quite difficult for me. So there was a lot of information on that part of the app about putting a rubber band around a lid (Participant 01, Female, aged 74).	app as useful self-management tool to diarise pain level and its association to everyday activities for persons with arthritis, some concerns highlight risk of pain monitoring preoccupation to a greater degree than necessary	useful self-management tool
Relevant to the user's self-management style and preferences. Participants suggested that a pain self-management app must be individualized to the specific type of arthritic pain so that the management strategies are tailored accordingly. … whether it's going to be rheumatoid arthritis, or whether it's going to be something else … you need to be able to have a button that says, "select your relevant (sic, arthritis)," … is yours osteo? is it rheumatoid? … (Participant 07, Male, aged 66). This suggestion extended beyond the overall orientation of the app, with participants perceiving that an app should have personalization features to suit the user's preferences.… it can't just be, like you said, a generic thing. Because people aren't generic (Participant 11, Female, aged 71).Participants indicated that the ideal arthritic pain app ought to include interactive video(s) of their personalized exercise regime, which they believed would act as a reminder, and ensure better compliance with the exercise instructions provided by their clinicians. The physio verbally gives me a list of exercises I should do. By the time I get home, of the five (sic exercises), I've only remembered two. So, having the video of an exercise which would remind me to do the five rather than just the two that I can remember, would be helpful (Participant 01, Female, aged 74).	app that include interactive videos for pain self-management tool needs to be individualized and tailored to specific type and stage of arthritis to suit users' preferences and ensure better compliance with exercise instructions,	users' preferences important
Participants indicated that these apps need to be personalized so that they took account of how long the user had experienced chronic pain, their baseline knowledge and skills level and then tailored the information and instructions accordingly. … I really felt that I had all of those skills under my belt at this stage. I believe that app would be a brilliant tool for someone in the early stages of their pain management. (Participant 09, Female, aged 76) Some participants mentioned that they felt no need to engage wish an app for their pain management needs, however were open to this approach out. I wouldn't see any need to have an app like that. I went to the app because I wanted to try something new (Participant 02, Female, aged76).	importance of personalization of apps meeting baseline knowledge and skills level with tailored information	tailored apps
Pain self-management apps must be designed with end-user ….While participants found the trial app to be relatively easy to use, they offered a range of suggestions on how an app could be more user friendly and helpful to them. Participants noted the challenges relating to vision and reading they faced when engaging with the app on a small screen. Yeah and if the graphs were such that you could actually see how you were going, that then that would perhaps help. I might be more likely to use it. (Participant 12, Female, aged 66); and I found the reading was beyond my capabilities. Too much writing and too small writing (Participant 19, Male, aged 72).	user friendly design regarding readable instructions and reminders to use app	manageable
It was apparent that a pain self-management app ought to include a peer engagement feature that would enable users to share their pain self-management experiences to better support one another … f you could check with other people because it's not always necessarily a lot of people placed around you who've got it (arthritis). You need to be talking to other people from here, there or other places (Participant 04,Female, aged 85).Furthermore, participants were also keen that the pain app ought to have an interactive push-notification feature to remind or prompt them to input their various assessment data. A little email reminder or a text reminder would be good. “Do your exercise”, or “Do your breathing”, or “Assess your pain and activity” … those kinds of things (Participant 04, Female, aged 85).	interactive push-notifications	reminders
Preferred management options Minimizing medical intervention and managing diabetes using lifestyle measures was clearly the first choice of approach. She wants to put me on this glycoside tablet to enhance whatever I've got in my body. I'm fighting against it. I says, I'll try and get my weight down. When my weight goes down, everything goes down, blood sugars and the blood pressure goes down (Patient 13, male, 60–64 years, Lothian) The diabetic nurse has picked up the phone twice to call me, looking at the results and to discuss a couple of things, so that was quite useful … It's actually led to a radical change in diet, following a discussion she had with our specialist diabetic GP (Patient 6, male, 50–54 years, Kent) However, for BP control, changes to medication initiated by professionals were mentioned more frequently. Yes, my diabetic practitioner nurse does call me about the readings, in fact she has modified my blood pressure control medication a couple of times to bring the levels down to a more acceptable level, so yeah, it's been a definite benefit I think(Patient 7, male, 55–59 years, Kent).	managing diabetes by lifestyle measures, weight loosing, and medication adjustment was defined as benefit	help to self-management
There was uncertainty about who should be responsible for initiating communication if readings were outside the target range. Some patients would wait for the practice nurse to contact them, which many did, others would initiate the communication themselves. But I don't know what to do if … I think that if it goes above 15, you have to do it again or something like that … I would let my practice get in touch with me, because I'm not very sure of what it all means. (Patient 20, male, 55–59 years Lothian) Well, I like to keep a weather eye on things. As for instance, I noticed that my blood pressure was not very high, but was in the high zone, and I brought that to their attention and said I was concerned, you know, that it should be lower. And as a consequence, they changed the medication, and it is now within the realms of being nearly normal now (Patient 8, female, 50–54 years, Kent).	patient self-observation led to changed medication	symptom awareness
. telemonitoring data was as a basis to change medical treatment. Frequent testing led to faster working through treatment protocols in some cases. However, as shown in the second example, not all professionals were willing to engage with the home monitoring data. I've got another gentleman that probably he needs to go on insulin. And we've, sort of, been watching whilst we've given him some tablets and they really haven't made any difference. So his treatment is going to change quicker than it would have done, sort of, normally. (Practice nurse 3) … from our point of view it's not really going to change what we'll do, I mean I'll change the medication based on her next HbA1c result rather than anything else ….it's potentially harmful to her to expect that blood glucose monitoring with home strips is actually useful when in reality we are just going to be keeping on checkingher HbA1c … but I mean who knows whether it's stimulated her thoughts and feelings about her diabetes and made her focus more on her diet and things (GP11) Some of these treatment changes were made without he need for the patient to visit the practice. I got a letter to say that it [BP] was a wee bit high, and I was to go and see a doctor, and he said, ‘Well come back in four weeks’ time,’ but … I didn't put on an appointment, because I was going away on holiday … So, by the time I come back there was a letter there, and they'd changed my medication. (Patient 20, male, 55–59 years, Lothian)	telemonitoring was a basis to change medical treatment without need to visit the practice	time saving treatment support

**Table 6:** Overview Themes and codes.

Themes	Welfare technology facilitates self-care and social contact	Welfare technology is perceived as helpful	Incorporating welfare technology into everyday life
**Codes**	Medical support app integration arthritic fingers impact use of app interactive push-notifications living with diabetes, constantly need to self-manage diabetes frequency of practitioners control symptom awareness telecare enables control and freedom in everyday life less dependency on visits from home care services and family caregivers essential support for its sustainable use technology made possible to keep contact with social networks WT window of opportunity to follow everyday activities in the world and maintain social life digital nursing home trust to sensors and that healthcare providers can identify if something is wrong reduction of home visits freedom of living alone for women and displeasure of living alone as male gender and view on loneliness scheduled videos are keeping exercise motivation in line, positive relationship with therapists body-worn fall detector sensor symptom monitoring and self-management of symptoms feeling of security with monitoring oxygen level and heath rate during exercise important early monitoring of acute exacerbation important for early treatment and prevention of serious condition constant monitoring as reminders for symptoms and security blanket	Helpful tool users' preferences important evidence-based/research- and context relevant apps user friendly design regarding readable instructions and reminders to use app patient involving time saving treatment support distance treatment fire and fall detectors for cognitive impairment safety and security fear as motivating factor to use WT sensor monitoring give sense of safety and reduce feeling of loneliness fall monitoring is important and detection of declined daily functions sensors as reminders on health problems and dependency, positive influence on exercise technology make possible not move from own homes and contribute to change of habits necessary for safety preferred technology that alarm directly ambulance education programme regarding digital devices gave opportunity to do exercise, social contact and skills to use iPad telerehabilitation afford more time with therapist and possibility to ask questions easier that face to face meeting reliable fall detection by automatic warning availability of contact person helping organisation of assistance real-time monitoring of heart rate, blood pressure, activity, sleep and coughing will prevent overdoing and taking of break or medications as well as understanding of symptoms	useful self-management tool tailored apps appreciated trying the system and contribution to research telecare is safe someone is watching me predictable independence reminders increase independence maintenance of independence and stay at home as long as possible individually adjusted education and support of WT skills important desiring to stay at home as long as possible even if WT is an alternative acceptance of sensors related to feeing of safety sensor monitoring as stimulation to exercises positive to know existence of welfare technology that could provide peace in mind technology that can withstand water and give exact location to emergency are desired telerehabilitation is time-saving and affordable specifically for those having difficulty to travel to local rehabilitation services opportunity to learn how use technology and accept technology education program of using electronic technology increased participants awareness of what it can be used for connection between body and feelings, better understanding of symptoms, secure doing of exercises good access to healthcare provider and health education as strengthening recognising and acceptance of illness, technology as help showing accurate status and reminders

## 3 Results

We found ten articles that met the inclusion criteria. Included studies were from the United Kingdom (n = 1) [26], Netherlands (n = 1) [30], Norway (n = 2) [27,31], Switzerland (n = 1) [33], Finland (n = 1) [28], Australia (n = 2) [25,32], and Canada (n = 2) [29,34]. All included studies used a qualitative design, and data was collected through interviews. In total, 161 old persons participated in included studies. WT in the included studies were apps, telemedicine, ICT, AAL, sensors, and wearable devices. Four studies stated that WT was used between 2 weeks and 6 months.

### 3.1 Welfare technology facilitates self-care and social contact

Most participants in the studies expressed a strong need to live in their own homes as long as possible and avoid ending up in a nursing home [[25], [26], [27], [28], [29], [30], [31], [32], [33], [34]]. This theme illustrates various aspects of how older participants expressed the impact of technology on self-care and alternatives to social contact. The older participants in the study said that apps help them better understand their health situation and plan their everyday activities. Living with chronic disease requires self-monitoring of symptoms. Apps provide more information about the signs of problems and what action to take if there is a change. Old adults with chronic diseases experience that WT leads to better follow-up of health status and medication adjustment to a greater extent. The WT was a contributing factor to independence by being used to manage practical everyday tasks such as medication reminders. Some older participants expressed that independent living was important to them, even if they had to get used to being monitored around the clock and everywhere, including bathrooms. One of the older participants highlighted that this type of monitoring could create some space for a feel-good form of resistance or play games with the system, such as opening the refrigerator to eat something to activate the monitoring system or turning on the shower without showering. At the same time, sensor monitoring provides a strong sense of security because someone is watching what is happening in the home space. The surveillance itself was perceived as an artificial form of company, which reduced feelings of loneliness. Another aspect highlighted was that monitoring equipment is perceived as discreet and does not require active involvement on the participant's part. These sensors are connected directly to healthcare providers. Most participants do not have any privacy concerns with sharing data with people they trust, especially if the data is the basis for assistance. For many, it is, therefore, easy to accept and trust around-the-clock in-home monitoring.

The old people participants described that observing their symptoms meant they could contact the care provider directly at short notice to discuss and possibly adjust prescriptions. This way, the treatment is maintained optimally, minimizing the risk of complications. They experienced a significantly shortened time between when a change in their health information was observed and when their medication was adjusted. At the same time, older patients experienced a feeling of empowerment. Another difference is that the introduction of WT reduces the number of booked visits to the care provider. All information about health status is registered in a patient's record, and it is easy for care providers to follow it up at a distance. Medication adjustment is smooth without requiring an appointment and travel time for a physical visit. In addition, a video link may provide close contact with the care providers.

### 3.2 Welfare technology is perceived as helpful

WT was perceived as an efficient aid to keep a good overview of disease symptoms. Such an overview makes it possible to plan medication and everyday activities [[25], [26], [27], [28], [29], [30], [31], [32], [33], [34]]. Some older users thought that they should be involved in WT design. In this way, they would have the opportunity to point out their needs as users that the software producers have not considered, for example, problems with vision, hearing, fine motor skills, or pain. This kind of involvement seems to be a way to empower older users. The use of telecare provides the opportunity to prevent accidents in the home, especially for people with a tendency to fall or cognitive impairment. Motion sensors and reminders are activated automatically. Further observation occurs at a distance, which is appreciated by the users, who do not want frequent visits by countless health personnel. Indoor and outdoor motion sensors, door alarms and personal alarms were perceived as providing a complete feeling of security. WTs are perceived as essential, and users should be offered training in all WT functions before they become an integral part of their lives. Older people's positive attitude towards WTs seems to be driven by fear of having an accident at home and not being able to contact anyone for help. WT solutions enable the older adults to continue living as they have before while knowing that someone will see it and can help if something happens. Regarding communication using WT, some people find it easier than others. The local context in which WT is used is also essential, including the care provider or institution to which it is linked. Users need to know where to turn when needed. User instructions must be accessible, understandable, and searchable, even for those with poor eyesight. Knowledge and skills related to WT have also been equated with citizens' rights. People who cannot manage these tools lose the opportunity to participate in planned activities, political processes, or private banking. While monitoring via distance undoubtedly gives a feeling of security, there is still ignorance about how this technology can reduce feelings of loneliness. Video meetings positively affect daily routines because people are motivated to look good in these meetings. Another aspect of WT is that it can capture things that users cannot note, such as changes in daily routines. Sensors that remind users about changes to their health can positively impact them and lead them to continue with prescribed exercises to maintain health and regain lost functions. Saving travel time to care institutions and providers is one of the much-appreciated positive benefits of using WT, which is why home rehabilitation using video conferencing is appreciated. Real-time monitoring of vital body parameters allows the user to feel calm. In such cases, the user can do his or her activity and rest.

### 3.3 Incorporating welfare technology into everyday life

The participants appreciated the opportunity to have contact with others in the same situation and be able to support each other [[25], [26], [27], [28], [29], [30], [31], [32], [33], [34]]. WT makes it possible to cope with everyday life, increases self-confidence, and makes life more meaningful by allowing people to follow what is happening in society. WT is essential for participating in modern life and maintaining social contact at a distance. It is easier to accept video surveillance if it allows one to continue living in one's own home and remain independent. For most older people, acceptance means integrating video surveillance as a natural part of life. WT, which consists of reminder sensors, primarily serves as a stimulus to perform exercises or move indoors and strengthens personal responsibility. Several people emphasized that they were interested in WT, which represents an excellent opportunity for them to remain in their homes because they cannot imagine moving. Many older adults had changed their views on WT and were willing to invest in it if necessary for their safety. WT provided a sense of security, allowing participants to live at home independently thanks to built-in sensors and the knowledge that if something happened, it would be discovered because there is someone on the other side of the technology who will note the need for help and respond to it.

## 4 Discussion of results

Results in this study illustrate that WT seems to be recognized and normalized among old adults because it enables them to stay in their homes. Furthermore, the technology allows them to have an overview of their health status and plan daily activities. They save time travelling to caregivers, it was easier for them to communicate online, and they dared to ask questions they would not have asked in person. Problems with loneliness and the need for human contact are not solved using WT. Chen's study [36] confirms that WT solutions often focus only on replacing or supplementing reduced physical needs to minimize risks. Users' emotional and psychological skills have been considered to a greater extent during the product development, design and implementation processes [36]. The implementation and use of WT became a must for many. Many had to discover some of the benefits, resulting in increased self-esteem [37]. Moreover, issues of inclusion, accessibility, digital literacy, and language were evident with old people users of WT. Saplacan and Herstad (2019) [38] discuss a case where robots were integrated into the homes of older people. Still, these people did not always understand the language used in the interaction design with the robots. In line with our results, Saplacan et al. (2021) [39] highlight the ethical challenges and opportunities resulting from integrating robots in-home care of older people. Challenges include the lack of legal frameworks and harmonized standards regulating artificial intelligence (AI) care systems and robots, decreased human contact, the feeling of objectivization and perceived loss of control over autonomy among older adults, aspects of privacy, deception, and infantilization as well as the problem of not knowing who is responsible for essential failures of the software or hardware used as part of the care process, the so-called many hands problem [39]. Similarly, Saplacan et al. [40] talk about the need for a sense of coherence. The WT needs to be understandable, manageable, and meaningful for older users: they should clearly understand how to use WT and for what purpose [41]. Other studies emphasize the need to carefully assess the utility value of the WT in the home care sector in opposition to privacy [42,43].

## 5 Strengths and limitations

There are several strengths of this research. The study protocol has been registered in advance in PROSPERO. The method was performed according to PRISMA guidelines. Published articles were identified in relevant databases using MeSH terms in close collaboration with the research librarian. Detailed information on selected studies' screening steps and quality control has been described in detail. However, the results can be seen in the light of limitations. The searches were limited to the period between 2015 and 2023. Therefore, it is possible that some relevant article was missed or that errors in the data collection were made. Inclusion criteria included studies published in English, which could result in missing research results in other languages.

## 6 Conclusion

The results of this study show the use of WT provides easier access to caregivers; it helps users develop a more profound knowledge of their disease and related symptoms, and being monitored at home offers a sense of security. In addition, it is necessary to increase WT literacy, especially among those who have not used the WT before. More studies on users and WT manufacturers and their interaction are needed.

## What this study adds

• This systematic literature review breaks new ground by focusing on old people as end users' subjective perceptions, evaluations, and interpretations of WT.
• This study shows that older people seem to accept WT if they feel that it provides an experience of security and independence in everyday life.

## Implications for policy and practice

• This study points to the end user's understanding, insight, and reasoning about what works well and what challenges remain regarding using WT. Developers of WT can use this information to address challenges by involving end users as co-creators.
• This study raises awareness of the potential of WT to support independent and autonomous ageing. Disseminating the results of this study can help create incentives for further research focusing on inclusive WT development. In this research, old people should be co-creating research subjects.

## CRediT author statement

The idea, conceptualization, methodology, supervision, analysis, results, writing-original draft preparation, co-writing of the original draft, reviewing, editing, and approving the final draft: Zada Pajalic (Professor Full, PhD, Dr Med Sci), Analysis, Results and Writing-Original draft preparation, co-writing original draft, Reviewing, Editing and Approving final draft: Gunilla Kulla (Associate professor, PhD), Co-writing original draft, Reviewing, Editing and Approving final draft: Sofia Elisabeth G. Olsen (RN, MSc), Annabel Hamre (RN, MSc), Benedicte Sørensen Strøm (Associate Professor, PhD), Celine Clausen (RN, MSc), Diana Saplacan (Senior Scientist, PhD).

## Declaration of competing interest

The authors declare that they have no known competing financial interests or personal relationships that could have appeared to influence the work reported in this paper.
